# Impact of Diagnostic Stewardship on Urine Culture Ordering in Saudi Arabia: Prospective Pre- and Postintervention Study

**DOI:** 10.2196/68044

**Published:** 2025-07-23

**Authors:** Ahlam Alghamdi, Afrah Alkazemi, Alnada Ibrahim, Mohammed Alraey, Mohammed Alaboud, Isra Farooqi, Mohammad Aatif Khan, Asem Allam, Mohammed Alwadai, Renad Alyahya, Ohoud Alzahrani, Hajar Y AlQahtani, Amir Mohareb, Muneerah Aleissa

**Affiliations:** 1 Department of Pharmacy Practice, College of Pharmacy, Princess Nourah bint Abdulrahman University, P.O. Box 84428, Riyadh, 11671, Saudi Arabia, 966 0118239313; 2 Department of Pharmacy Practice, College of Pharmacy, Kuwait University, Kuwait City, Kuwait; 3 Department of Infectious Diseases, King Abdullah bin Abdulaziz University Hospital, Riyadh, Saudi Arabia; 4 Microbiology Laboratory, Department of Pathology and Laboratory Medicine, King Abdullah Bin Abdul-Aziz University Hospital, Riyadh, Saudi Arabia; 5 Pharmaceutical Care Services, King Abdullah bin Abdulaziz University Hospital, Riyadh, Saudi Arabia; 6 College of Pharmacy, Princess Nourah bint Abdulrahman University, Riyadh, Saudi Arabia; 7 Department of Pharmaceutical Care, Ministry of National Guard Health Affairs, Riyadh, Saudi Arabia; 8 King Abdullah International Medical Research Center, Riyadh, Saudi Arabia; 9 King Saud Bin Abdul-Aziz University for Health Sciences, Riyadh, Saudi Arabia; 10 Division of Infectious Diseases, Massachusetts General Hospital, Boston, MA, United States

**Keywords:** urine culture, diagnostic stewardship, Saudi Arabia, automation, asymptomatic bacteriuria, antimicrobial stewardship program

## Abstract

**Background:**

Inappropriate testing of urine cultures can lead to overuse of antibiotics, antimicrobial resistance, *Clostridioides difficile* infections, and increased cost. In Saudi Arabia, antimicrobial stewardship programs have improved antibiotic use but lack focus on asymptomatic bacteriuria. Targeted interventions are needed to address this gap.

**Objective:**

We assessed the implementation of a clinical decision support (CDS) tool in diagnostic stewardship, focusing on the appropriateness of urine culture orders and antibiotic use.

**Methods:**

We examined differences in urine culture testing and antibiotic use before and after implementation of a CDS tool in a 400-bed hospital in Riyadh, Saudi Arabia, from August 2021 to July 2022. We included adult patients with urine culture orders. Our outcomes were the percentage of urine cultures ordered that were inappropriate and antibiotic use after the implementation of the CDS intervention. We used a multivariable logistic regression model to determine factors associated with inappropriate urine culture testing and antibiotic use.

**Results:**

The percentage of inappropriate urine culture orders were significantly lower in the postintervention period compared to the preintervention period (821/2254, 36.4% vs 754/1814, 41.6%; *P*=.001). The CDS intervention was associated with 16.7% lower odds of inappropriate urine culture ordering (adjusted odds ratio [aOR] 0.83, 95% CI 0.73‐0.95; *P*=.008). Unnecessary antibiotics were significantly lower in the postintervention period (310/2254, 72.9% vs 288/1814, 85.7%; *P*<.001). The CDS intervention was associated with a 52% reduction in unnecessary antibiotic use (aOR 0.487, 95% CL 0.332‐0.713; *P*<.001)

**Conclusions:**

A CDS initiative can reduce unnecessary urine culture testing and antibiotic overuse.

## Introduction

Urinary tract infections (UTIs) are common in both the inpatient and outpatient settings [1,2]. Despite being common, appropriate diagnosis and use of urine cultures remain a challenge. Inappropriate urine culture testing, for example, screening for asymptomatic bacteriuria (ASB), can be the first step in a chain of events that can lead to antibiotic overuse, antimicrobial resistance (AMR), *Clostridioides difficile* infections, and increased health care cost [3]. Antimicrobial therapy is not recommended in most cases of ASB, and withholding treatment has no major adverse outcomes in most cases [4,5]. Current Infectious Diseases Society of America (IDSA) guidelines recommend screening for and treating ASB only in pregnant women or individuals undergoing invasive urologic procedures [6]. Despite these recommendations, ASB remain one of the most common causes of antimicrobial overuse, where up to 80% of patients with ASB are treated with antibiotics [7].

Challenges in the clinical differentiation between ASB and UTIs may offer a partial explanation for the excessive overtreatment of ASB [8]. Diagnostic stewardship of urine cultures can play an important role in reducing downstream overuse of antibiotics [7]. While many studies have evaluated stewardship of urine culture testing in Europe and North America, research is limited in other geographic settings [9]. For example, there are limited data about diagnostic stewardship of urine cultures from the Eastern Mediterranean region, which is a major research gap given the rising prevalence of AMR in that region [10]. In Saudi Arabia, antimicrobial stewardship programs (ASPs) have been successfully implemented to optimize antibiotic use and reduce antimicrobial resistance but have, thus far, generally lacked a focus on addressing antibiotic use associated with ASB [11]. Therefore, we sought to assess the impact of a clinical decision support (CDS) intervention on diagnostic stewardship, focusing on the appropriateness of urine culture orders and antibiotic use.

## Methods

### Overview

We conducted a quasiexperimental study at an academic medical center with 400 beds in Riyadh, Saudi Arabia. Our center is a referral center for women’s health, pediatrics, and adolescent health. We compared clinical practice in a preintervention phase (August 2021 to January 2022) and a postintervention phase (February 2022 to July 2022) without incorporating a washout period, in both the inpatient and outpatient settings. We included adult patients (≥18 years old) with urine culture orders during the study period and excluded patients on antibiotics prescribed for infections prior to the urine culture and those with missing key data such as UTI signs and symptoms, laboratory results, and clear antibiotic indication.

### Endpoints and Definitions

We assessed the impact of our CDS initiative on diagnostic stewardship through measuring inappropriate urine culture and antibiotic use. Appropriate practice for ordering a urine culture, as approved by the international guideline and institutional ASP committee, includes documentation of signs or symptoms of a UTI, pregnancy, and patients who are undergoing invasive urologic procedures, immunocompromised, or critically ill [6]. Therefore, we defined inappropriate urine culture testing in circumstances where patients had no documented signs or symptoms of a UTI, pregnancy, urologic procedures, immunosuppression, or critical illness [9]. Signs and symptoms of a UTI include dysuria, suprapubic pain, flank pain, costovertebral angle tenderness, septic shock, and altered mental status in older (≥65 years) patients [12]. Antibiotics prescribed for ASB without any of the approved indications listed above were deemed to be unnecessary.

### Diagnostic Stewardship Interventions

**Figure 1.**
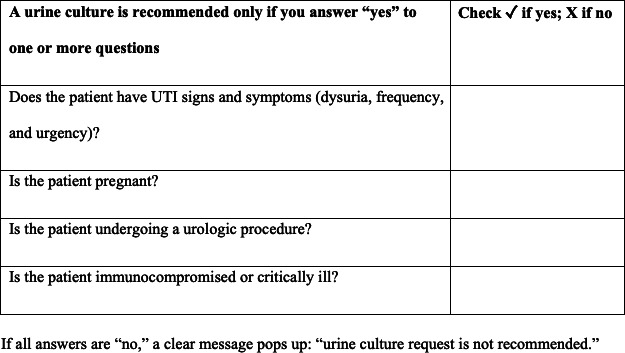
Urine culture request–directed questions implemented in the electronic health record. UTI: urinary tract infection.

We implemented the institutional ASP–approved CDS in electronic health records (EHRs) on February 1, 2022. The goal of this initiative is to serve as a voluntary guide for physicians to appropriately request urine cultures in the outpatient and inpatient settings. Prior to requesting any urine culture, appropriate urine request–directed questions are asked (Figure 1). If all answers are “no,” an alert is triggered indicating that a urine culture may not be recommended for this patient. Physicians are able to override the CDS and still order a urine culture even if the alert is triggered. Alongside the CDS initiative’s rollout, the ASP committee disseminated best practices related to UTI care, including indications for urine cultures, to all health care providers.

### Statistical Analysis

We analyzed categorical data using a chi-square test or Fisher exact test, as appropriate, and continuous data using a Student *t* test. We also used a multivariable logistic regression model, adjusting for age, sex, diabetes mellitus, and chronic kidney disease, to evaluate the association between our CDS intervention and inappropriate urine culture orders and unnecessary antibiotic use. All analyses were conducted in STATA/BE (version 18; StataCorp).

### Ethical Considerations

Ethical approval was obtained from the Institutional Review Board of King Abdullah bin Abdul-Aziz University Hospital (IRB log number: 22‐0005). As this was a retrospective study, informed consent was not required. The study protocol was reviewed and approved by the institutional review board, which granted a waiver of informed consent in accordance with national and institutional ethical guidelines. All data were deidentified prior to analysis. Data were handled securely in compliance with institutional data protection policies to ensure participant confidentiality.

## Results

**Figure 2.**
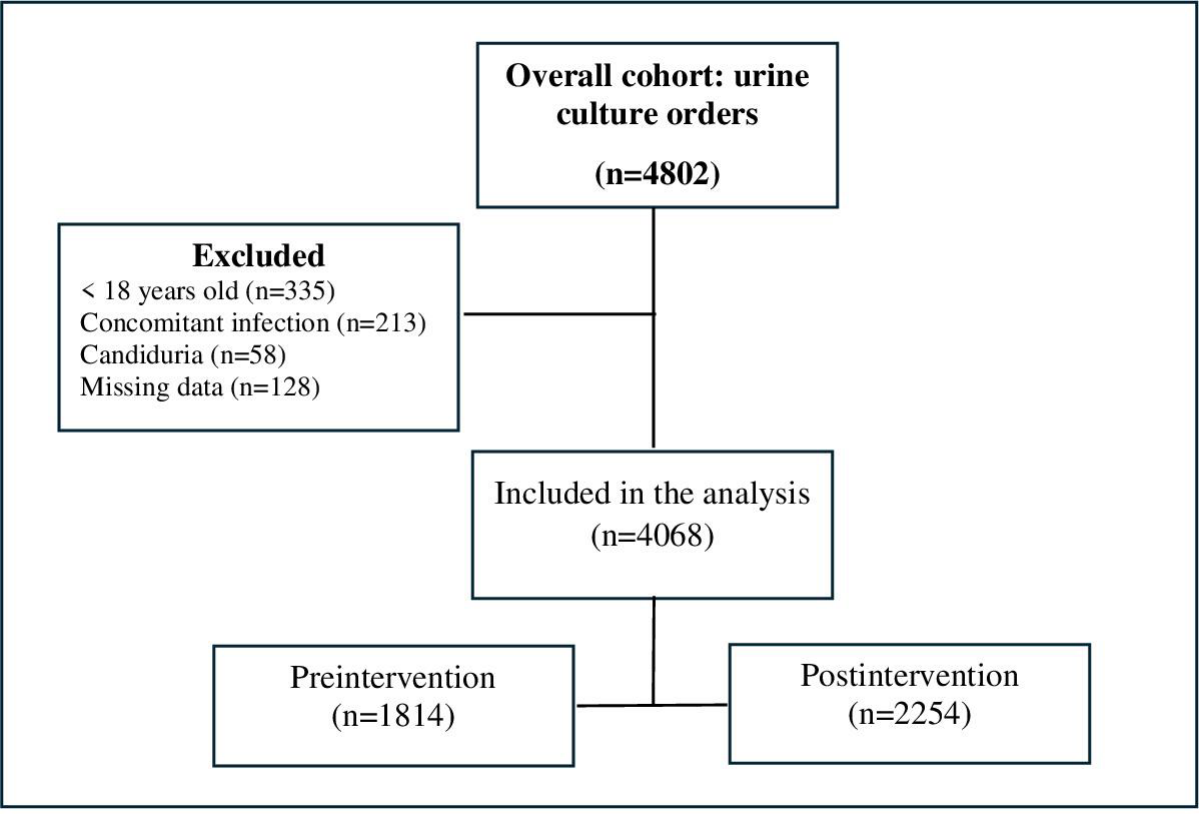
Flow diagram of study participants who had urine culture orders.

During the study period, we identified 4068 urine culture orders among 3846 unique patients, of which 1814 were ordered in the preintervention period and 2254 in the postintervention period (Figure 2). Demographic information about the study participants is shown in Table 1. The mean age was 42.2 (17.40) years and the majority were female (3068/4068, 75.4%), which is consistent with our clinical setting. Approximately 33% (1375/4068) of patients were pregnant, 4.4% (180/4068) underwent urologic procedures, 4.4% (181/4068) were immunocompromised, and 1.8% (75/4068) were critically ill. Overall, 10.6% (435/4068) had a positive urine culture, of which 32.9% (n=143) were symptomatic and 67.1% (n=292) were asymptomatic (Table 1).

Inappropriate urine culture orders, per our stewardship initiative criteria, were significantly lower in the postintervention period compared to the preintervention period (821/2254, 36.4% vs 754/1814, 41.6%, respectively; *P*=.001). The CDS intervention was associated with a 20% reduction in inappropriate urine culture orders (odds ratio [OR] 0.805; 95% Cl 0.71‐0.91; *P*=.001). In a multivariable logistic regression adjusting for possible confounders, the CDS intervention was associated with 16.7% lower odds of inappropriate urine culture ordering based on our ordering criteria (adjusted odds ratio [aOR] 0.83; 95% CI 0.73‐0.95; *P*=.008; Table 2).

Additionally, we found that 1097 antibiotic regimens were prescribed: 336 in the preintervention period and 761 in the postintervention period. The use of unnecessary antibiotics was significantly lower in the postintervention period versus the preintervention period (310/2254, 72.9% vs 288/1814, 85.7%, respectively; *P*<.001). Our CDS intervention was associated with a 55% reduction in the unnecessary antibiotic use (OR 0.449; 95% CI 0.309‐0.65; *P*<.001). In a multivariable logistic regression adjusting for possible confounding variables, the CDS intervention was associated with a 52% reduction in unnecessary antibiotic use (aOR 0.487; 95% CI 0.332‐0.713; *P*<.001; Table 2).

**Table 1.** Characteristics of patients who had urine culture orders (N=4068) in the preintervention and postintervention periods, Saudia Arabia, August 2021 to July 2022.

	Preintervention (n=1814)	Postintervention (n=2254)	*P* value
Age (years), mean (SD)	42.04 (17.42)	42.42 (17.46)	.50
Sex (female), n (%)	1254 (69.1)	1814 (80.5)	<.001
Medical comorbidities, n (%)			
Heart disease	122 (6.7)	135 (6)	.34
Diabetes mellitus	369 (20.3)	442 (19.6)	.56
Hypertension	294 (16.2)	398 (17.7)	.22
Chronic kidney disease	74 (4.1)	51 (2.3)	.001
Liver disease	6 (0.3)	8 (0.4)	.90
Respiratory disease	133 (7.3)	223 (9.9)	.004
Active malignancy	8 (0.4)	15 (0.7)	.34
Pregnancy, n (%)	627 (34.6)	748 (33.2)	.36
Urologic procedure, n (%)	18 (1)	162 (7.2)	<.001
Immunosuppressant agent, n (%)	35 (1.9)	146 (6.5)	<.001
ICU^a^ at index culture, n (%)	47 (2.6)	28 (1.2)	<.001
Urine culture characteristics, n (%)			
Positive urine culture	165 (9.1)	270 (12)	.003
UTI^b^	24 (14.5)	119 (44)	<.001
ASB^c^	141 (85.5)	151 (56)	.19

a ICU: intensive care unit.

b UTI: urinary tract infection.

c ASB: asymptomatic bacteriuria.

**Table 2.** Outcomes of the clinical decision support (CDS) initiative at pre- and postintervention using logistic regression model.

Outcome	Unadjusted odds ratio (OR)		Adjusted odds ratio (aOR)^a^	
	OR (95% CI)	*P* value	aOR (95% CI)	*P* value
Inappropriate urine culture orders post- versus preintervention	0.805 (0.710‐0.914)	<.001	0.83 (0.73‐0.95)	.008
Unnecessary antibiotic use post- versus preintervention	0.449 (0.309‐0.652)	<.001	0.487 (0.332‐0.713)	<.001

a Adjusting for age, sex, diabetes mellitus, and chronic kidney disease.

## Discussion

### Principal Findings

To our knowledge, this is the first study to evaluate a diagnostic stewardship initiative targeting inappropriate urine culture orders and unnecessary antibiotic use in patients with UTIs and ASB in Saudi Arabia. Numerous stewardship programs have been implemented in various geographic regions to reduce unnecessary antibiotic use for ASB [13]. Some of these initiatives include continuous education, pocket cards, audit and feedback, and computer-based reminders [13]. In this study, we used a CDS tool embedded in EHRs to guide the physicians to appropriately order urine cultures, which could lead to a reduction in unnecessary antibiotic use. The CDS initiative we implemented successfully decreased inappropriate screening for ASB as measured by the decrease in urine culture orders. Similarly, our initiative also successfully decreased ASB overtreatment as measured by the decrease in unnecessary antibiotic use. While antibiotic prescriptions were numerically higher in the postintervention phase compared to the preintervention phase, the percentage of unnecessary antibiotics was significantly lower in the postintervention phase. The higher number of antibiotics prescribed is likely due to the fact that the postintervention phase comprised a higher proportion of patients who underwent urologic procedures or were immunocompromised. By implementing a CDS tool to address inappropriate urine culture orders and unnecessary antibiotic use in patients with ASB, our study contributes directly to efforts aimed at optimizing antimicrobial stewardship in Saudi Arabia. The rise of AMR is a global public health crisis, associated with rising morbidity, mortality, and health care costs [14]. Single-center reports suggest a high prevalence of multidrug-resistant organisms, which complicate patient care and threaten gains made from other public health advances in the region [15,16]. In Saudi Arabia, as in many international settings, antimicrobial use is not well regulated and injudicious use of antibiotics is a major factor for AMR development [17]. Recent studies demonstrate that inappropriate antibiotic use in the management of UTIs is approximately 50%, with the primary reason being ASB [18,19]. In our study, the use of unnecessary antibiotics decreased by 52%, highlighting the value of our CDS tool to potentially aid in reducing the rate of AMR in Saudi Arabia.

Several studies have explored other diagnostic stewardship interventions aimed at reducing unnecessary antimicrobial therapy for UTIs and ASB. A recent study that was done in Hong Kong found that suppressed urine culture results led to a 23% reduction in antimicrobial use [20]. Similarly, another study implemented an initiative of not reporting positive urine culture results unless requested via telephone, which led to a decrease in antimicrobial use by 36% [21]. These findings collectively support the effectiveness of targeted interventions in optimizing diagnostic practices and antimicrobial stewardship. By reducing the number of unnecessary urine cultures, health care providers can reduce inappropriate antibiotic use, thus aligning with efforts to combat AMR and improve patient care outcomes.

Our study has limitations. First, as this was a retrospective analysis of a quality improvement project, the results are possibly susceptible to confounding factors. While we accounted for several factors influencing antimicrobial use in our multivariable regression model, these findings should be further confirmed in controlled prospective studies. Future studies should specifically evaluate outpatient versus inpatient urine culture ordering, which we were not able to do through our form of data collection. Moreover, our study may not be generalizable to other hospitals in the region, given that it was a single-centered study. Finally, although the assessments of unnecessary antibiotics were conducted by infectious disease specialists, they were not formally validated, potentially leading to discrepancies between evaluators.

### Conclusions

In summary, we implemented an ASP-approved CDS initiative to optimize urine culture testing and antimicrobial use and to reduce overtreatment of ASB. This initiative resulted in a 16.7% reduction in urine culture testing and a 52% reduction in antimicrobial use for UTIs. This is one of the few studies of such initiatives from the Eastern Mediterranean region, which is a site of rising AMR.

## Abbreviations

AMR: antimicrobial resistance
aOR: adjusted odds ratio
ASB: asymptomatic bacteriuria
ASP: antimicrobial stewardship program
CDS: clinical decision support
EHR: electronic health record
IDSA: Infectious Diseases Society of America
OR: odds ratio
UTI: urinary tract infection
